# Safety and effectiveness of carbon nanoparticles suspension-guided lymph node dissection during thyroidectomy in patients with thyroid papillary cancer: a prospective, multicenter, randomized, blank-controlled trial

**DOI:** 10.3389/fendo.2023.1251820

**Published:** 2024-01-08

**Authors:** Jingzhu Zhao, Junyi Wang, Ruochuan Cheng, Jianwu Qin, Zhilong Ai, Hui Sun, Zhuming Guo, Xiaohua Zhang, Xiangqian Zheng, Ming Gao

**Affiliations:** ^1^ Department of Thyroid and Neck Tumor, Tianjin Medical University Cancer Institute and Hospital, National Clinical Research Center for Cancer, Tianjin’s Clinical Research Center for Cancer, Key Laboratory of Cancer Prevention and Therapy, Tianjin, China; ^2^ Department of Thyroid Disease Diagnosis and Treatment Center, First Affiliated Hospital of Kunming Medical University, Yunnan, China; ^3^ Department of Thyroid & Neck Surgery, Henan Cancer Hospital, Affiliated Cancer Hospital of Zhengzhou University, Zhengzhou, China; ^4^ General Surgery, Zhongshan Hospital Fudan University, Shanghai, China; ^5^ Department of Thyroid Surgery, China Japan Union Hospital of Jilin University, Jilin, China; ^6^ State Key Laboratory of Oncology in South China, Collaborative Innovation Center for Cancer Medicine, Sun Yat-sen University Cancer Center, Guangzhou, China; ^7^ Department of Thyroid & Breast Surgery, The First Affiliated Hospital of Wenzhou Medical University, Wenzhou, China; ^8^ Department of Breast and Thyroid Diseases, Tianjin Union Medical Center, Tianjin, China

**Keywords:** clinical trial, papillary thyroid cancer, carbon nanoparticles, parathyroid hormone, lymph node, thyroidectomy

## Abstract

**Objective:**

This study aimed to evaluate the effectiveness and safety of carbon nanoparticles**-**guided lymph node dissection during thyroidectomy in patients with papillary thyroid cancer(PTC).

**Methods:**

Clinical trials consisted of two subgroups: unilateral lobectomy (UL; n=283) and total thyroidectomy (TT; n=286). From each subgroup, the patients were randomly assigned to two groups: the carbon nanoparticle group and control group. Primary endpoints included parathyroid hormone (PTH) levels, number of lymph nodes (LNs) detected, number of tiny lymph nodes detected, and recognition and retention of the parathyroid glands. Secondary endpoint was recognition and protection of the recurrent laryngeal nerve.

**Results:**

**A** total of 569 patients with PTC were recruited. There were no statistically significant differences in demographics between the carbon nanoparticles and control groups (P > 0.05). In the UL subgroup, there were no significant differences in PTH levels between the two groups at preoperative, intraoperative, and postoperative day one, and postoperative month one (P>0.05). There was no significant difference in the serum Ca^2+^ levels between the two groups preoperatively and at postoperative month one (P>0.05). The number of lymph nodes dissected in the carbon nanoparticles group was significantly higher than that in the control group (P<0.0001). The detection rate of tiny lymph nodes in the carbon nanoparticles group was higher than that in the control group (P=0.0268). In the TT subgroup, there was no significant difference in PTH levels between the two groups at preoperative, intraoperative, and postoperative day one (P>0.05). However, the mean PTH level in the carbon nanoparticles group was significantly higher than that of the control group at postoperative month one (P=0.0368). There was no significant difference in the serum Ca^2+^ levels between the two groups preoperatively and at postoperative month one (P>0.05). There were no significant differences between the two groups in the number of dissected LNs (P>0.05) or the detection rate of tiny lymph nodes (P>0.05). No drug-related AE and complications due to the injection of carbon nanoparticles were recorded in this study. There were no significant differences between the two groups in terms of parathyroid preserved *in situ* and recurrent laryngeal nerve injury in the UL and TT subgroups.

**Conclusions:**

Carbon nanoparticles demonstrated efficacy and safety in thyroidectomy. The application of carbon nanoparticles could significantly facilitate the identification and clearance of LNs and the optimum preservation of parathyroid function.

**Clinical trial registration:**

https://www.chictr.org.cn/, identifier ChiCTR2300068502.

## 1 Background

Papillary thyroid cancer (PTC) is a common endocrine malignancy, and its incidence has increased substantially in recent decades (1). The most effective treatment for PTC is complete removal of the primary tumor and metastatic regional lymph nodes. Complete lymph node dissection is key to reducing recurrence and improving prognosis. However, the patient’s quality of life needs to be considered (2). Hypoparathyroidism and voice disorders are the two most common complications that can considerably affect the quality of life of patients (3). Effective protection of the parathyroid gland and recurrent laryngeal nerve is the best way to improve a patient’s quality of life.

Carbon nanoparticles (CNs) are novel lymphatic tracers that have been successfully used for lymph node tracing in colorectal, breast, and gastric cancers (4–6). CNs have the advantages of a high specificity in the lymphatic system and low toxicity. Recently, carbon nanoparticles have been used to detect lymph nodes in PTC (7, 8). However, they were all monocentric studies with limited sample sizes. We conducted this prospective multicenter randomized controlled trial to evaluate the safety and effectiveness of carbon nanoparticles in thyroidectomy for PTC.

## 2 Patients and methods

### 2.1 Study design

This was a multicenter, randomized, blank-controlled study (ChiCTR2300068502 Http://www.chictr.org.cn/). This study was conducted in accordance with the Declaration of Helsinki and approved by the institutional review board. Written informed consent was obtained from all patients.

Based on the different resection ranges of the primary lesions, the clinical trials were divided into two subgroups: unilateral lobectomy and total thyroidectomy.

Patients satisfying the following criteria were recruited in the unilateral lobectomy group: (1) preoperative and intraoperative diagnosis of untreated, unilateral, and single-focal papillary thyroid carcinoma; (2) no cervical lymph node metastasis on imaging examination, and no indication of lateral neck lymph node dissection; (3) unilateral lobectomy plus isthmectomy with ipsilateral central lymph node dissection; (4) age 18–70 years; (5) results of preoperative laboratory blood test–neutrophils (ANC) ≥1.5×10^9^/L, platelets (PLT) ≥100×10^9^/L, total bilirubin (TBI) ≤2 times the upper limit of normal (ULN) (2 mg/dL), alanine aminotransferase (ALT) ≤2 times the ULN, and aspartate aminotransferase (AST) ≤2 times the ULN; and (6) signed informed consent.

Patients who met the following criteria were recruited into the total thyroidectomy group: (1) preoperatively and intraoperatively diagnosed with untreated, unilateral, and single-focal papillary thyroid carcinoma; (2) no enlarged lymph nodes on physical examination; and lateral cervical lymph node metastasis on imaging examination; (3) total/near total thyroidectomy, ipsilateral central and lateral lymph node dissection; (4) age 18–70 years; (5) preoperative laboratory blood test results (ANC≥1.5×10^9^/L, PLT≥100×10^9^/L, TBI ≤ 2 times ULN (2 mg/dL), ALT ≤ 2 times ULN, AST ≤ 2 times ULN; and (6) signed informed consent.

Patients were excluded if they met one or more of the following criteria: (1) concomitant Hashimoto’s thyroiditis; (2) active stage of liver disease or abnormal liver function, with ALT, AST, and TBIL≥2 times the ULN; (3) renal function impairment, Cr≥2 times the ULN or BUN≥2 times the ULN; (4) a history of alcohol, drug, or substance abuse and disturbance of blood coagulation; and (5) a history of serious uncontrolled medical disease, recent myocardial infarction, or acute infection.

### 2.2 Outcome

The primary endpoints included parathyroid hormone (PTH) levels, number of lymph nodes detected, number of tiny lymph nodes detected, and recognition and retention of the parathyroid glands. The secondary endpoint was recognition and protection of the recurrent laryngeal nerve. PTH levels were measured preoperatively, intraoperatively, and on postoperative days 1 and 1 month.

### 2.3 Statistical methods

All statistical analyses were performed using SAS version 9.4 statistical professional analysis software. Measurements were reported as the mean standard deviation. Continuous measurements were compared using the t-test, and count data were analyzed using the chi-squared test. The Wilcoxon rank-sum test was used for intergroup comparisons. The safety analysis was based on descriptive analytics. The adverse events and the incidence in each group were listed in the table and compared using the chi-square test or Fisher’s exact test. Statistical significance was set at P value < 0.05.

## 3 Results

### 3.1 Patients’ characteristics

**Figure 1:**
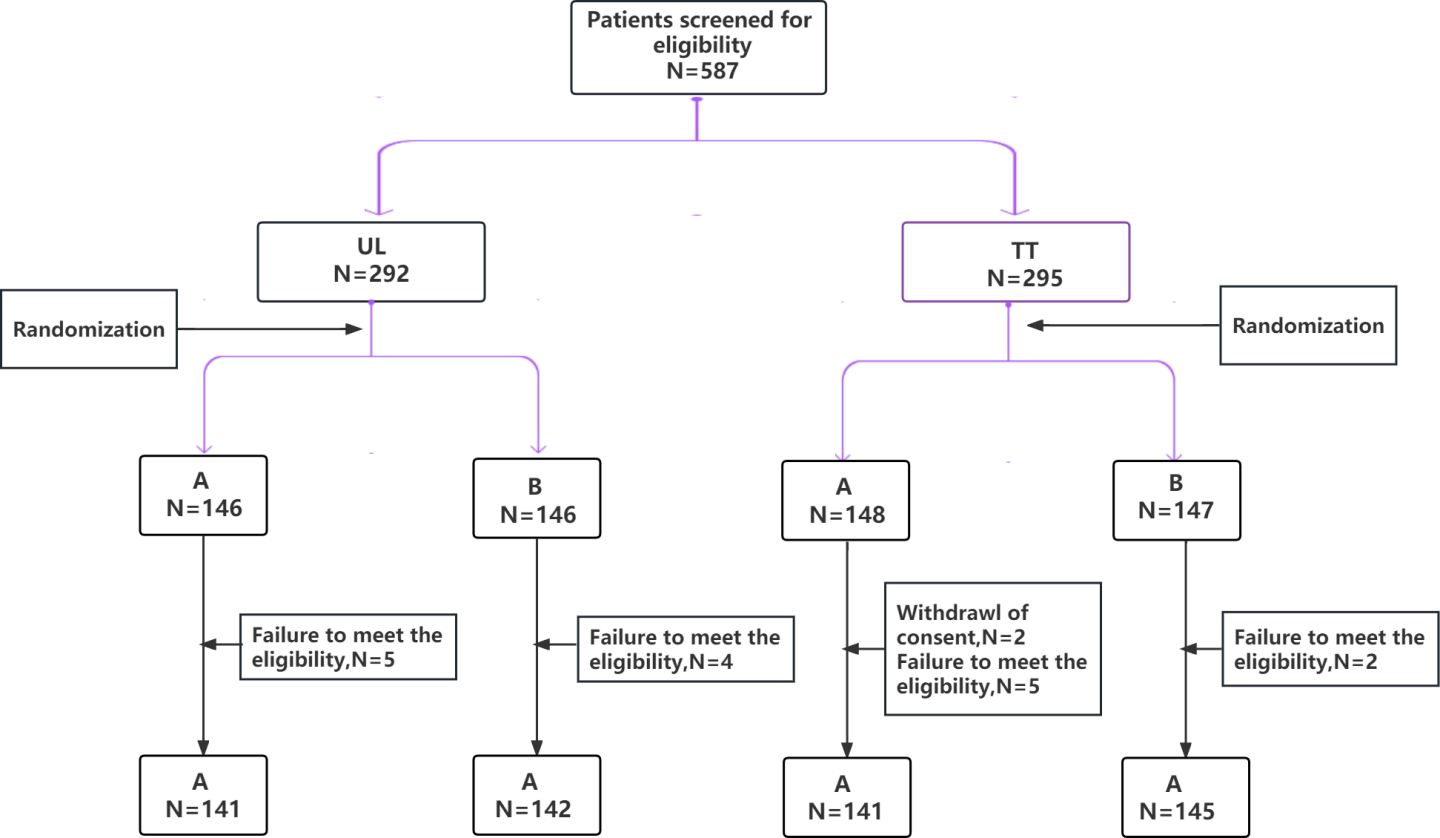
CONSORT diagram of patients randomized to used carbon nanoparticles group (A group) or control group (B group). According to the different resection ranges of the primary lesion, they were assigned to two subgroups: the unilateral lobectomy group (UL) and total thyroidectomy group (TT).

**Figure 2:**
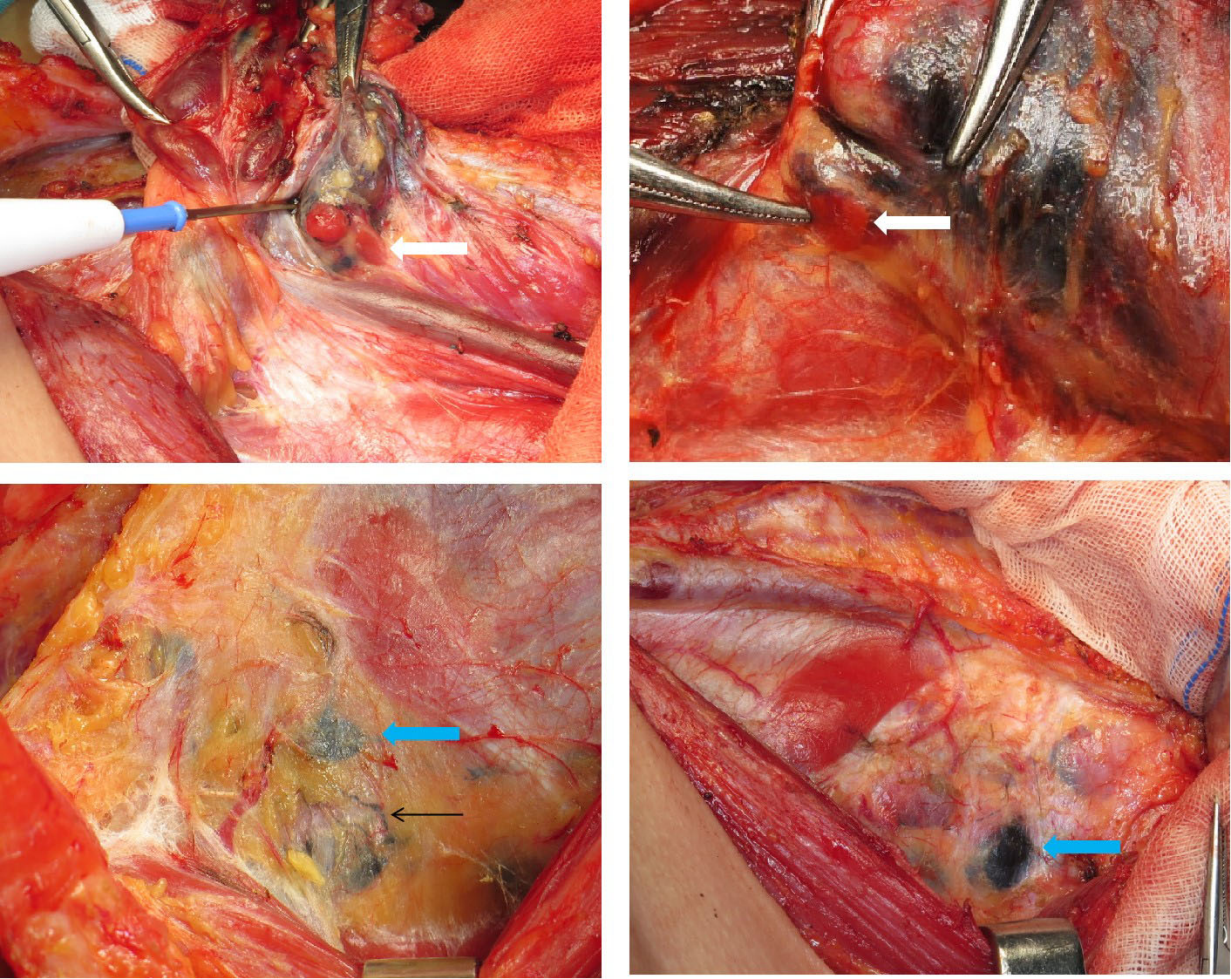
Carbon nanoparticles were injected into the thyroid around the tumour; the white arrow shows the parathyroid gland(negative staining); the green arrows show the lymph nodes coloured black; the black arrows show the lymph ducts coloured black.

A total of 569 patients with PTC from seven hospitals across China were enrolled. According to the different resection ranges of the primary lesion, they were assigned to two subgroups: the unilateral lobectomy group (UL, 283 patients) and total thyroidectomy group (TT, 286 patients), who were randomly assigned to the carbon nanoparticles group (A group) or control group (B group), respectively (**Figure 1**). The LNs in the A group were stained black by nanoparticles (**Figure 2**). As shown in **Table 1**, no significant differences were detected between the two groups regarding baseline characteristics.

**Table 1:** Comparison of baseline characteristics between carbon nanoparticles group and control group in UL and TT subgroup.

		UL		TT	
		A(N=141)	B(N=142)	A(N=141)	B(N=145)
Gender					
	Female	93(65.96)	100(70.42)	90(63.83)	95(65.52)
	Male	48(34.04)	42(29.58)	51(36.17)	50(34.48)
	χ^2^	0.65		0.09	
	*P*	0.4200		0.7653	
Ages(Y)					
	Mean ± SD	41.17 ± 9.96	42.50 ± 10.66	40.16 ± 10.57	37.80 ± 10.48
	Median	42.00	42.00	40.00	37.00
	Min~Max	21.00~66.00	21.00~69.00	17.00~68.00	14.00~65.00
	t	1.084		1.90	
	*P*	0.2794		0.0586	
BMI(kg/m2)					
	Mean ± SD	23.70 ± 3.56	23.65 ± 3.36	23.78 ± 3.42	25.11 ± 4.12
	Median	23.2	23.43	23.50	24.60
	Min~Max	16.20~34.60	16.53~31.20	17.57~33.00	17.58~41.50
	t	0.136		2.31	
	*P*	0.8917		0.0208	
Allergic history					
	No	123(87.23)	120(84.51)	126(89.36)	12(86.21)
	Yes	18(12.77)	22(15.49)	15(10.64)	20(13.79)
	χ^2^	0.43		0.66	
	*P*	0.5103		0.4157	
Stage T	N(missing)	132(9)	133(9)	132(9)	142(3)
	T1+T2	111(84.09)	111(83.46)	105(79.55)	105(73.94)
	T3+T4	21(15.91)	22(16.54)	27(20.45)	37(26.06)
	χ^2^	0.02		1.20	
	*P*	0.8890		0.2735	
Stage N	N(missing)	131(10)	133(9)	132(9)	142(3)
	NX	0(0.00)	4(3.01)	0(0.00)	0(0.00)
	N0	81(61.83)	82(61.65)	4(3.03)	8(5.63)
	N1	50(38.17)	47(35.34)	128(96.97)	134(94.37)
	CMH*-*χ^2^	4.07		1.11	
	*P*	0.1308		0.2927	

BMI, Body mass index; A, carbon nanoparticles group; B, Control group.

### 3.2 Changes and comparison of PTH level

In the UL group, there were no significant differences between the carbon nanoparticles and control groups in terms of preoperative (P=0.1582), intraoperative (P=0.9048), and postoperative day one (P=0.7747) and first month (P=0.3840) serum PTH levels, **Table 2**.

**Table 2:** Changes and comparison of parathyroid hormone at different time points in UL and TT subgroup.

		UL			TT	
		A(N=141)	B(N=142)		A(N=141)	B(N=145)
Preoperative	N(missing)	135(6)	137(5)	N	134(7)	142(3)
	Mean(sd)	37.91(21.99)	34.52(17.23)	Mean(sd)	29.51(23.26)	28.60(24.28)
	Median	36.09	34.6	Median	28.32	29.80
	Min~max	2.18~164.41	2.30~81.90	Min~max	1.02~123.60	1.20~114.80
	t	1.42		Wilcoxon Z	0.40	
	*P*	0.1582		*P*	0.6870	
Intraoperative	N(missing)	140(1)	137(5)	N	140(1)	143(2)
	Mean(sd)	32.65(19.92)	32.95(20.97)	Mean(sd)	11.97(14.48)	13.41(19.46)
	Median	28.92	31.4	Median	6.04	5.82
	Min~max	2.50~97.40	2.10~120.00	Min~max	0.38~76.46	0.29~115.50
	t	0.12		Wilcoxon Z	0.42	
	*P*	0.9048		*P*	0.6719	
Postoperative days 1	N(missing)	138(3)	135(7)	N	137(4)	143(2)
	Mean(sd)	25.94(15.63)	25.40(15.04)	Mean(sd)	8.79(9.82)	8.76(11.24)
	Median	27.13	25.9	Median	5.39	3.93
	Min~max	0.90~71.30	1.70~64.40	Min~max	0.01~53.40	0.10~61.44
	t	0.29		Wilcoxon Z	0.80	
	*P*	0.7747		*P*	0.4255	
Postoperative 1 month	N(missing)	125(16)	130(12)	N	121(20)	133(12)
	Mean(sd)	35.08(16.23)	37.07(20.01)	Mean(sd)	25.73(19.80)	21.09(19.55)
	Median	34.5	34.43	Median	24.88	17.16
	Min~max	1.31~79.70	2.50~132.70	Min~max	1.29~82.20	0.60~80.17
	t	0.87		Wilcoxon Z	2.09	
	*P*	0.3840		*P*	0.0368	

A: Carbon nanoparticles group; B: Control group.

In the TT group, there was no significant difference between the carbon nanoparticles and control groups in terms of preoperative (P=0.6870), intraoperative (P=0.6719), and postoperative day one (P=0.4255) serum PTH levels. However, the mean serum PTH in carbon nanoparticles group was significantly higher than that of the control group (25.92 ± 19.76 mmol/L *vs*. 21.29 ± 19.68 mmol/L, P=0.0368) on first month post-operation, **Table 2**.

### 3.3 Comparison of serum Ca^2+^ level

In the UL group, there was no significant difference between the carbon nanoparticles and control groups in terms of preoperative (P=0.4237) and first month post-operation (P=0.428) serum Ca^2+^ levels. While, there was no significant difference between the carbon nanoparticle and control groups in terms of preoperative (P=0.6148) and first month post-operation (P=0.99) serum Ca^2+^ levels in the TT group, **Table 3**.

**Table 3:** Comparison of serum Ca^2+^ level between carbon nanoparticles group and control group in UL and TT subgroup.

		UL			TT	
		A(N=141)	B(N=142)		A(N=141)	B(N=145)
Preoperative	Mean(sd)	2.33(0.11)	2.32(0.12)	Mean(sd)	2.35(0.11)	2.36(0.20)
	t	0.8		t	0.50	
	*P*	0.4237		*P*	0.6148	
Postoperative 1 month	Mean(sd)	2.36(0.12)	2.40(0.48)	Mean(sd)	2.36(0.16)	2.35(0.18)
	K-W	0.63		Wilcoxon Z	0.00	
	*P*	0.428		*P*	0.99	

A: Carbon nanoparticles group; B: Control group.

### 3.4 Comparison of the number of LNs dissected

In UL group, the number of LNs dissected from the carbon nanoparticles group was significantly higher than that of the control group (8.02 ± 7.70 vs 5.37 ± 5.63, P< 0.0001). The detection rate of tiny lymph nodes in the carbon nanoparticles group was higher than that in the control group (P=0.0268). The number of tiny lymph nodes was also higher in the carbon nanoparticles group; however, the difference was not statistically significant (4.99 ± 3.80 vs. 3.93 ± 3.23, P=0.0823). Furthermore, significantly more ≤2 mm lymph nodes were detected in the carbon nanoparticles group than that of the control group (2.45 ± 3.14 vs. 1.45 ± 2.55, P=0.0034), **Table 4**.

**Table 4:** Comparion of the number of LNs dissected between carbon nanoparticles group and control group in UL and TT subgroup.

		UL		TT	
		A(N=141)	B(N=142)	A(N=141)	B(N=145)
Number of LNs dissected	N(missing)	139(2)	142(0)	136(5)	144(1)
	Mean(sd)	8.02(7.70)	5.37(5.63)	40.82(21.61)	38.20(19.14)
	Median	6	4	36.00	35.00
	Min~max	0.00~70.00	0.00~35.00	9.00~139.00	6.00~152.00
	Wilcoxon Z	4.71		0.78	
	*P*	<0.0001		0.4346	
Tiny LNs	N(missing)	139(2)	140(2)	136(5)	144(1)
	Yes(%)	76(54.68)	58(41.43)	109(80.15)	113(78.47)
	No(%)	63(45.32)	82(58.57)	27(19.85)	31(21.53)
	χ^2^	4.9		0.12	
	*P*	0.0268		0.7296	
Number of tiny LNs	N(missing)	76(0)	58(0)	109(0)	113(0)
	Mean(sd)	4.99(3.80)	3.93(3.23)	12.15(8.96)	10.45(7.73)
	Median	4	3	9.00	9.00
	Min~max	1.00~17.00	1.00~20.00	1.00~44.00	1.00~34.00
	Wilcoxon Z	1.74		1.36	
	*P*	0.0823		0.1750	
Number of ≤2mm LNs	N(missing)	138(3)	138(4)	141(7)	144(3)
	Mean(sd)	2.45(3.14)	1.45(2.55)	9.77(9.34)	8.24(7.96)
	Median	1	0	1	0
	Min~max	0.00~16.00	0.00~20.00	0.00~44.00	0.00~34.00
	Wilcoxon Z	2.93		1.16	
	*P*	0.0034		0.2449	

LNs: lymph nodes; A: Carbon nanoparticles group; B: Control group.

In the TT group, the number of dissected LNs was higher in the carbon nanoparticles group; however, the difference was not statistically significant (40.82 ± 21.61 vs. 38.20 ± 19.14, P=0.4346). In addition, the numbers and detection rate of tiny lymph nodes was also higher in the carbon nanoparticles group; however, the difference was not statistically significant (80.15% vs. 78.47%, P=0.7296;12.15 ± 8.96 vs 10.45 ± 7.73, P=0.1750). Furthermore, the number of ≤2 mm lymph nodes was also higher in the carbon nanoparticles group, and the difference was not statistically significant (9.77 ± 9.34 vs 8.24 ± 7.96, P=0.2449), **Table 4**.

### 3.5 Safety outcomes and operative complications

No drug-related AE or complications due to the injection of carbon nanoparticles were recorded in this study. There were no significant differences between the two groups in recurrent laryngeal nerve injury (three cases *vs*. one case) in the UL group. There were no significant differences between the two groups in recurrent laryngeal nerve injury (five *vs*. four cases) in the TT group. There were no significant differences between the carbon nanoparticles group and control groups regarding the parathyroid gland preserved *in situ* in the UL and TT groups, **Table 5**.

**Table 5:** Comparion of parathyroid preserved *in situ* between carbon nanoparticles and control groups in UL and TT subgroup.

	UL		TT	
	A(N=124)	B(N=125)	A(N=124)	B(N=133)
Upper-left				
N(missing)	53(71)	61(64)	123(1)	127(6)
Yes	49(92.45)	61(100.00)	119(96.75)	123(96.85)
No	4(7.55)	0(0.00)	4(3.25)	4(3.15)
P*	0.0439		1.0000	
Upper-right				
N(missing)	71(53)	65(60)	123(1)	132(1)
Yes	67(94.37)	62(95.38)	120(97.56)	126(95.45)
No	4(5.63)	3(4.62)	3(2.44)	6(4.55)
P*	1.0000		0.5025	
Lower-left				
N(missing)	53(71)	61(64)	112(12)	126(7)
Yes	42(79.25)	52(85.25)	99(88.39)	105(83.33)
No	11(20.75)	9(14.75)	13(11.61)	21(16.67)
χ^2^	0.71		1.24	
*P*	0.4008		0.2656	
Lower-right				
N(missing)	71(53)	65(60)	117(7)	129(4)
Yes	65(91.55)	51(78.46)	105(89.74)	104(80.62)
No	6(8.45)	14(21.54)	12(10.26)	25(19.38)
χ^2^	4.63		4.00	
*P*	0.0313		0.0456	

*Fisher exact test; A: Carbon nanoparticles group; B: Control group.

## 4 Discussion

This is the first prospective multicenter randomized blank-controlled trial. The study met its primary endpoint and carbon nanoparticles were proven to be safe.

The most effective treatment for PTC is complete removal of the primary and metastatic lymph nodes. However, the insensitivity of preoperative imaging may lead to an incomplete lymph node dissection. Although not usually fatal, it can lead to repeated surgeries and more damage to patients. Previous sample- or single-center studies have shown that carbon nanoparticles can effectively detect more lymph nodes in colorectal cancer (6, 9) and papillary thyroid cancer (7, 8, 10). In contrast, another study showed that carbon nanoparticles did not generate significant benefits in lymph node dissection (11). However, this study shows that CNs may help detect more lymph nodes and tiny lymph nodes in papillary thyroid cancer, which may allow for more radical dissection. But, in terms of the number of lymph nodes removed, the trend between the UL groups and TT groups was inconsistent, which may be due to the following reasons: 1. Due to dynamic reasons, carbon nanoparticles are more likely to distribute in the central lymph nodes, and the distribution is limited in the whole region of the lateral neck, and the number of lymph nodes in the lateral neck region is much more than that in the central region. 2. In the TT group, patients with lateral cervical metastasis had a high probability of lymphatic blockage, and carbon nanoparticles were not easily distributed in lymphatic vessels.

Post-thyroidectomy hypocalcemia is the most common complication that seriously affects patients’ quality of life. In a few cases, hypocalcemia can be severe and lead to acute life-threatening conditions, such as tetany, laryngospasm, arrhythmias, and heart failure (12). Effective preservation of the parathyroid gland is the best method to prevent hypocalcemia.

Clinically, parathyroid glands may be accidentally removed as LNs owing to variations in position. CNs have high specificity for the lymphatic system as lymph tracers. CNs have been successfully applied in gastric (13) and breast cancers (14). Once entering the lymphatic system, CNs can cause black-stained of the LNs but not neighboring tissues, especially the parathyroid gland, so-called “negative staining”. This allows for the identification and clearance of LNs and the optimum preservation of parathyroid function. In our study, although there was no significant difference in the number of preserved parathyroid glands between the carbon nanoparticles and control groups, the parathyroid hormone level recovered more quickly in the carbon nanoparticles group than in the control group within the postoperative one month, indicating that carbon nanoparticles are essential for the protection of parathyroid function. This apparent protection by CNs staining may help surgeons preserve the parathyroid glands *in situ* during the operation with maximum retention of the blood supply, including tiny blood vessels around the parathyroid gland. Therefore, carbon nanoparticles are recommended for patients undergoing thyroidectomy, particularly for total thyroidectomy.

Although there was a significant difference in the parathyroid hormone level between the carbon nanoparticles and control groups at postoperative one month in the TT subgroup, there was no significant difference in serum Ca^2+^ levels, possibly because patients were given regular calcium supplements.

No drug-related AE or complications due to the injection of carbon nanoparticles were recorded in this study. There were no significant differences between the two groups with respect to recurrent laryngeal nerve injury in the UL and TT subgroups. None of the patients experienced any discomfort during the study period. The clinical application of CNs has shown no toxicity (6, 13, 15).

This study had several limitations. First, this study could not be double-blind and may be biased when clinicians perform surgery. More, this study adopted competitive enrollment, and experienced surgical centers have more participants, which may lead to selection bias. Experienced surgeons, whether using carbon nanoparticles or not, have less impact on parathyroid blood flow and are less likely to miscut.

## 5 Conclusion

In conclusion, this prospective, multicenter, randomized, blank-controlled study confirmed that CNs are effective and safe for papillary thyroid cancer. More importantly, the application of carbon nanoparticles could significantly facilitate the identification and clearance of LNs and optimum preservation of parathyroid function.

## Data availability statement

The original contributions presented in the study are included in the article/supplementary material. Further inquiries can be directed to the corresponding authors.

## Ethics statement

This study was conducted in accordance with the Declaration of Helsinki and approved by the institutional review board. Written informed consent was obtained from all patients. The studies were conducted in accordance with the local legislation and institutional requirements. Written informed consent for participation in this study was provided by the participants’ legal guardians/next of kin.

## Author contributions

Literature research and article preparation by JZ. Data collection and statistical analysis by JW. Clinical studies, study concepts, and design by RC, JQ, ZA, HS, ZG, XZha, XZhe and MG. The corresponding author had full access to the data and took final responsibility for the decision to submit for publication. All authors contributed to the article and approved the submitted version.
